# Assessing the Factors of User Resistance to Adopting Telehealth Technology Among Women

**DOI:** 10.1177/26924366251380141

**Published:** 2025-09-24

**Authors:** Israt Jahan Shithii

**Affiliations:** Department of Management Information Systems, Noakhali Science and Technology University, Noakhali, Bangladesh.

**Keywords:** telehealth adoption, user resistance, women, Bangladesh

## Abstract

**Background:**

Despite the growing global shift toward digital health solutions, the adoption of telehealth technologies in Bangladesh remains a concerning issue. Psychological factors such as lack of digital literacy, trust, and familiarity may significantly hinder the uptake of telehealth, particularly in developing countries where digital integration in health care is still evolving.

**Objective:**

This study aims to identify and evaluate the psychological barriers—specifically lack of familiarity (LOR), lack of digital literacy (LDL), and lack of trust (LT)—that influence user resistance (UR) to adopting telehealth technologies in Bangladesh. Furthermore, women among different age groups have been used as an independent construct to explore how age influences the resistance to adopting telemedicine technology.

**Methods:**

One hundred and fifty respondents were administered a structured questionnaire based on a Likert scale. Constructs were measured using items validated through factor analysis. Reliability was confirmed via Cronbach’s alpha. Multiple regression analysis was conducted to test the hypothesized relationships between psychological factors (LOR, LDL, LT) and UR.

**Results:**

The model explained 47.6% of the variance in UR (*R*^2^ = 0.476, *p* < 0.001). Both LDL (β = 0.359, *p* < 0.001) and LT (β = 0.410, *p* < 0.001) were found to be significant predictors of resistance. However, LOR (β = 0.078, *p* = 0.201) did not significantly influence UR. All constructs demonstrated strong internal consistency (Cronbach’s α > 0.76). The Kruskal–Wallis test indicated that age significantly affects women’s resistance to adopting telemedicine. Subsequent pairwise comparisons among age groups revealed that older women exhibit higher levels of resistance compared with their younger counterparts.

**Conclusion:**

The findings suggest that enhancing digital skills and building trust are critical to reducing resistance and promoting the adoption of telehealth in Bangladesh. While unfamiliarity alone does not deter adoption, it becomes a barrier when compounded by low digital literacy and trust concerns. Targeted interventions such as digital literacy training and transparent communication about data security could facilitate greater acceptance and integration of telehealth technologies in the country.

## Introduction

The global health care system is undergoing a significant transformation with the rise of telehealth technologies, including virtual doctor consultations, remote patient monitoring, and online health management platforms.^1^ The adoption of telehealth increases medical access, improves service efficiency, and enhances overall patient management outcomes, especially in developing countries with limited health care facilities. In Bangladesh, telehealth has emerged as a potential solution to challenges such as limited access to medical professionals, disparities between urban and rural health care facilities, and inadequate health care infrastructure.^2^ Despite its considerable advantages, the adoption of telehealth technologies in Bangladesh has been slow and uneven.

One of the main reasons for this slow adoption is user resistance (UR)—the reluctance or unwillingness to use new digital health tools or systems. To address this issue and increase the uptake of telehealth, it is essential to understand the psychological factors behind UR, such as fear of technology, lack of knowledge, or lack of trust (LT) in the system.^3^ The innovation resistance theory provides a useful framework for understanding why users may resist adopting new technologies,^4^ particularly telehealth systems.

However, studies have highlighted age as a crucial factor impacting acceptance and resistance to health technology, particularly telemedicine. Older persons frequently show increased reluctance due to variables such as lower digital literacy, less experience with digital platforms, and a stronger dependence on traditional health care approaches.^5,6^ However, younger people are more adaptive and open to telehealth, most likely because they have had more exposure to technology and have more comfort in using digital tools.^7,8^ These age-related discrepancies indicate that resistance to telemedicine is not consistent across age groups, which supports the reason for incorporating age as a major variable in this research. Studies have also shown that women may face unique challenges and varying levels of resistance to adopting telemedicine.^9^ Women’s adoption of health technologies is influenced by multiple interrelated factors, including digital literacy, perceived usefulness, trust in technology, and sociocultural roles.^10^ Women, particularly in middle to older age groups, often report lower confidence in using digital platforms, which contributes to increased hesitation and resistance.^8^

This study focuses on three key psychological factors—lack of familiarity (LOR), lack of digital literacy (LDL), and LT—which are hypothesized to influence UR to adopting telehealth technologies in Bangladesh. These psychological barriers are particularly relevant in Bangladesh, where a large portion of the population is not accustomed to using digital tools, and where concerns about trust in technology and data privacy are widespread. The different age groups within women are considered to find the impact of age as a factor on the resistance behavior to adopt telemedicine technology in Bangladesh.

### LOR and UR

Previous research has shown that users with limited experience with digital health platforms are more likely to feel overwhelmed and uncertain when asked to interact with them.^10,11^ This unfamiliarity can lead to feelings of discomfort and increased resistance to adoption. Users who are unfamiliar with telehealth technologies may struggle to navigate digital health tools, leading to frustration and reluctance to incorporate them into their health care routines (Moore & Benbasat, 1991). This aligns with the hypothesis that LOR positively influences UR, as unfamiliarity with telehealth technologies can exacerbate resistance to their adoption.^12^

### LDL and UR

Digital literacy refers to the ability to use digital tools (such as smartphones, computers, and online platform) competently and confidently.^13^ Individuals without these skills are more likely to resist using telehealth technologies, such as online consultations, health apps, or hospital websites. Prior studies have shown that low levels of digital literacy correlate with increased anxiety and confusion when using digital systems.^14^ In the context of telehealth, a lack of digital skills can create barriers to effective interaction with telehealth systems, leading to greater resistance to adoption.^15^ It is hypothesized that an LDL will positively influence UR to telehealth adoption.

### LT and UR

The psychological factor of LT in telehealth systems, particularly in terms of concerns about data security, diagnostic reliability, and privacy, plays a significant role in the resistance to telehealth adoption.^16^ Research consistently shows that trust is a crucial factor in the acceptance of new technologies, especially in sensitive areas such as health care.^17^ Users who perceive risks related to data privacy, the accuracy of digital diagnoses, or the potential misuse of personal health information are less likely to adopt telehealth technologies.^18^ Trust in the technology and its providers is essential for overcoming resistance and fostering adoption.^19^ Therefore, it is hypothesized that an LT will positively influence UR to adopting telehealth technologies.

In summary, the prior research has shown that psychological factors, such as familiarity, trust, and digital literacy, significantly influence the adoption of new technologies. In Bangladesh, these factors are particularly important due to socioeconomic and infrastructural challenges. LOR, LDL, and LT are key psychological barriers that impact UR to telehealth adoption. While telehealth holds immense potential to improve health care delivery in Bangladesh,^20^ these psychological factors can prevent people from embracing digital health solutions. As health care in Bangladesh becomes increasingly digitized, understanding how these barriers impact adoption and developing strategies to address them is critical. The findings from this study will provide valuable insights into the key psychological barriers to telehealth adoption in Bangladesh and offer practical recommendations for overcoming these barriers.

### Hypotheses

The study aims to test the following hypotheses to assess the influence of these psychological factors on UR to adopting telehealth technologies in Bangladesh. The different age groups of women have also been considered to find the impact on resistance behavior of telemedicine adoption.

Hypothesis 1: LOR with telehealth technologies positively influences UR to adopting telehealth.

Hypothesis 2: LDL positively influences UR to adopting telehealth.

Hypothesis 3: LT in telehealth systems positively influences UR to adopting telehealth.

Hypothesis 4: There is a significant difference in resistance to telemedicine across age groups of women.

## Materials and Methods

### Research design

This study used a quantitative, cross-sectional research design to assess the psychological factors influencing UR to adopting telehealth technologies. The key psychological barriers examined include LOR, LDL, and LT. The study aimed to identify how these factors impact UR toward telehealth platforms and determine the significance of each factor in shaping users’ willingness to adopt telehealth solutions. This study also focuses on how women within different age groups may vary in resistance behavior to adopt telemedicine technology.

### The operational definition of variables

In this study, four key constructs related to the resistance to telemedicine adoption among women are examined.

LOR is defined as the extent to which women are unfamiliar with the concept, features, and processes of telemedicine systems.^21^ This includes individuals who might express sentiments such as “I don’t know what telemedicine is.”

LDL refers to the degree to which women have a limited ability or an inability to use digital tools such as smartphones, computers, or health-related applications.^22^ For example, a respondent might report, “I struggle to use telemedicine even if willing, due to a lack of technical skills.”

LT captures women’s doubts or concerns about the privacy, security, credibility, or diagnostic accuracy of telemedicine platforms,^23^ as reflected in statements such as “I don’t trust video consultations.”

UR is the extent to which women intentionally avoid or oppose telemedicine technologies.^24^ For example, “I don’t want to deal with telemedicine.”

These operational definitions guide the development and interpretation of the survey instruments used in this study.

### Participants

A convenience sampling method was employed to recruit participants for the study. The sample consisted of 150 women from various demographic backgrounds. Participants were selected based on their familiarity with and experience using telehealth platforms, with inclusion criteria focusing on adults aged 18 and above.

Given the exclusive focus on women who resist telemedicine technology adoption may vary significantly across different life stages. Thus, participants were categorized into age groups to allow for co-group analysis.

### Ethical approval

This study complied with the ethical principles outlined in the Declaration of Helsinki.^25^ It was considered exempt from institutional review board review, and the requirement for informed consent was waived as participation was voluntary, anonymous, and posed minimal risk to participants as per U.S. Department of Health and Human Services, 2018. Informed consent was obtained from all participants before their inclusion in the study. Participants provided written permission for using their data in the publication of this research. Ethical approval was obtained from the ethical committee, Noakhali Science and Technology University (Ref: NSTU/FBS/EC/2025/81).

### Data collection

Data were collected using a structured questionnaire consisting of Likert-scale items. The items were designed to measure the key psychological barriers to adopting telehealth systems, specifically focusing on:

- **LOR:** Measured through three items related to the user’s familiarity with telehealth tools and their comfort level in using digital platforms for health care.
- **LDL:** Assessed using three items measuring the participants’ perceived competence in using telehealth technologies and their need for training or assistance.
- **LT:** Evaluated using five items addressing concerns regarding data privacy, security, and the reliability of telehealth platforms.
- **UR:** Measured through four items assessing the participants’ resistance to adopting telehealth systems, their preference for traditional health care, and their perceived need for in-person consultations.

The questionnaire was pretested for clarity and reliability. Respondents were asked to rate each item on a 5-point Likert scale ranging from 1 (strongly disagree) to 5 (strongly agree).

### Statistical analysis

Factor analysis was conducted to examine the underlying dimensions of the constructs (LOR, LDL, LT, and UR). Principal component analysis (PCA) was used as the extraction method, and the suitability of the data for factor analysis was evaluated using the Kaiser–Meyer–Olkin (KMO) measure and Bartlett’s test of sphericity. Reliability of the constructs was assessed using Cronbach’s alpha.

Multiple regression analysis was performed to examine the relationship between the independent variables (LOR, LDL, LT) and the dependent variable (UR). The model was assessed for its fit using the *R*-squared value, which indicates how much of the variation in the dependent variable (UR to telehealth) can be explained by the independent variables (LOR, LDL, and LT). The significance of the regression model was confirmed using analysis of variance (ANOVA), and individual coefficients were assessed to determine the contribution of each predictor to UR. The hypothesis testing was conducted using *t*-tests for the regression coefficients. Statistical significance was set at a *p* value of 0.05.

This age-based grouping enabled a Kruskal–Wallis test to determine whether resistance to telemedicine significantly varied across age categories within women. Again, to identify specific differences between these age groups, a pairwise comparisons were conducted. These analyses provide a deeper understanding of how women’s resistance to telemedicine technology may be influenced by age-related factors.

## Result

### The demographic profile of women participants

A total of 150 women participated in the study. The age distribution was as follows: 6% were aged 18–25 years, 13.5% were 25–30 years, 28% were 30–35 years, 36% were 35–40 years, and 16.5% were above 40 years. In terms of education, 13.5% of participants had education below SSC, 27.5% had completed SSC, 36.5% had completed HSC, 18% had an honors degree, and 5.5% held a master’s degree. Regarding occupation, 2% of participants were students, 37.5% were housewives, 15.5% worked in public service, 11.5% were in private service, 19.5% were engaged in business, and 14% reported other occupations (Table 1).

**Table 1.** Demographic Profile

	Total	Percentile
Gender		
Women	150	100
Age		
18–25	09	6
25–30	20	13.5
30–35	42	28
35–40	54	36
40 above	25	16.5
Education		
Below SSC	19	13.5
SSC	41	27.5
HSC	55	36.5
Honors degree	27	18.0
Master’s degree	08	5.5
Occupation		
Student	03	2.0
Housewife	56	37.5
Public service	23	15.5
Private service	17	11.50
Business	30	19.50
Other	21	14.0

### Sample adequacy test for factor analysis

#### KMO and Bartlett’s test results

The KMO measure of sampling adequacy was found to be 0.884, indicating that the sample was suitable for factor analysis. Bartlett’s test of sphericity yielded a chi-square value of 1524.275 with 171 degrees of freedom and was statistically significant (*p* < 0.001), supporting the suitability of the data for structure detection (Table 2).

**Table 2.** Kaiser–Meyer–Olkin and Bartlett’s Test

Kaiser–Meyer–Olkin measure of sampling adequacy	0.884
Bartlett’s test of sphericity	
Approximate chi-square	1524.275
df	171
Significance	0.000

This is very high (above 0.80), which falls into the “meritorious” category.^26^ This means the dataset is highly suitable for factor analysis. In the Bartlett’s test, the *p* value (0.000) is highly significant, meaning the null hypothesis can be rejected as the correlation matrix is an identity matrix. In other words, the variables are correlated enough to proceed with factor analysis.

### PCA: Communalities

Communalities were assessed using PCA as the extraction method. The results showed that the majority of variables had moderate to high communalities after extraction, indicating that they were well represented by the factor solution. Specifically, *LOR1*, *LOR2*, and *LOR3* had extracted communalities of 0.713, 0.694, and 0.684, respectively. The variables *lt1* through *lt5* showed communalities ranging from 0.361 (*lt5*) to 0.730 (*lt3*), with *lt5* having the lowest extraction value among all items (Table 3).

**Table 3.** Principal Analysis Component Communalities

	Initial	Extraction
lof1	1.000	0.713
lof2	1.000	0.694
lof3	1.000	0.684
lt1	1.000	0.648
lt2	1.000	0.635
lt3	1.000	0.730
lt4	1.000	0.656
lt5	1.000	0.361
ldl1	1.000	0.530
ldl2	1.000	0.608
ldl3	1.000	0.709
ldl4	1.000	0.695
ur1	1.000	0.607
ur2	1.000	0.747
ur3	1.000	0.590
ur4	1.000	0.604

Extraction method: Principal component analysis.

For the *ldl* variables, the communalities ranged from 0.530 (*ldl1*) to 0.709 (*ldl3*). The *ur* variables also demonstrated good representation in the factor solution, with extraction values of 0.607 (*ur1*), 0.747 (*ur2*), 0.590 (*ur3*), and 0.604 (*ur4*). These results indicate that the selected items are adequately explained by the extracted components (Table 3).

Communalities values indicated that most variables had moderate to high common variance explained by the extracted components, with extraction values ranging from 0.590 to 0.747. The item *lt5* showed a lower communality value (0.361), suggesting it may not align well with the underlying factor structure, but as it is not below 0.30, it is still considered in the analysis process.^27^

### Internal consistency test

#### Internal consistency reliability

Cronbach’s alpha was calculated to assess the internal consistency of the constructs used in the study. The results indicated acceptable reliability for all constructs. The LOR construct had a Cronbach’s α value of 0.796, while LDL yielded a value of 0.774. The LT construct showed an α value of 0.787, and UR had a reliability coefficient of 0.760 (Table 4). These values suggest that each construct demonstrated a satisfactory level of internal consistency for further analysis.

**Table 4.** Cronbach’s Alpha Test for Internal Consistency

Construct	α values
LOR	0.796
LDL	0.774
LT	0.787
UR	0.760

LOR, lack of familiarity; LDL, lack of digital literacy; LT, lack of trust; UR, user resistance.

The internal consistency test was conducted using Cronbach’s alpha to assess the reliability of the constructs used in the study: LOR, LDL, LT, and UR. All constructs demonstrated acceptable levels of internal consistency, with α values exceeding the commonly accepted threshold of 0.70 (2). Specifically, LOR had the highest reliability with an α value of 0.796, followed closely by LT at 0.787 and LDL at 0.774, indicating that the items within each of these constructs are consistently measuring the same underlying concept. The dependent variable UR also showed good reliability with an α value of 0.760. These results suggest that the measurement scales used for all constructs in the study are reliable and internally consistent.

### Regression analysis summary

A multiple regression analysis was conducted to examine the influence of LOR, LDL, and LT on UR. The model demonstrated a strong relationship with an *R* value of 0.690 and an *R*^2^ of 0.476, indicating that approximately 47.6% of the variance in UR can be explained by the predictors. The adjusted *R*^2^ was 0.468, with a standard error of the estimate of 0.65091 (Table 5).

**Table 5.** Regression Analysis Model Summary

Model	*R*	*R* ^2^	Adjusted *R*^2^	Standard error of the estimate	Change statistics				
					*R*^2^ change	*F* change	df1	df2	Significance *F* change
1	0.690^a^	0.476	0.468	0.65091	0.476	59.394	3	196	0.000

^a^ Predictors: (Constant), LOR, LDL, and LT.

Dependent variable: UR.

LOR, lack of familiarity; LDL, lack of digital literacy; LT, lack of trust; UR, user resistance.

The model showed statistical significance, with an *F* change of 59.394, degrees of freedom (df1 = 3, df2 = 196), and a significance level of *p* < 0.001 (Table 5). These results confirm that the predictors collectively have a significant impact on UR to telehealth adoption.

The regression model aims to examine the relationship between the dependent variable UR and the independent variables LOR, LDL, and LT. The model shows a multiple correlation coefficient (*R*) of 0.690, indicating a moderately strong positive correlation between the predicted and actual values of UR. The coefficient of determination (*R*^2^) is 0.476, meaning that approximately 47.6% of the variance in UR can be explained by the independent variables included in the model. The adjusted *R*^2^ value of 0.468 slightly adjusts for the number of predictors, confirming a good model fit without overfitting. The standard error of the estimate is 0.65091, which indicates the average distance between the observed and predicted values of UR. The *F*-test result is statistically significant (*F* = 59.394, *p* < 0.001), suggesting that the overall model is a good fit and that the independent variables together significantly predict the dependent variable UR.^28^

### ANOVA results

An ANOVA test was conducted to assess the overall significance of the regression model, examining the effects of LOR, LDL, and LT on UR. The analysis revealed that the regression model was statistically significant, with a regression sum of squares of 75.492, a residual sum of squares of 83.041, and a total sum of squares of 158.533.

The model yielded an *F*-value of 59.394 with 3 and 196 degrees of freedom, and the significance level was *p* < 0.001, indicating that the predictors collectively explained a significant portion of the variance in UR.

The ANOVA table provides further confirmation of the overall significance of the regression model predicting UR from the independent variables LOR, LDL, and LT. The regression sum of squares is 75.492 with 3 degrees of freedom, representing the variation in UR explained by the model. The residual sum of squares is 83.041 with 196 degrees of freedom, indicating the variation that remains unexplained by the model. The total sum of squares is 158.533, which is the total variation in the dependent variable UR. The mean square for regression is 25.164, and the mean square for residuals is 0.424. The *F*-statistic is 59.394, and the corresponding *p* value is 0.000, which is statistically significant (*p* < 0.001) (Table 6). This result indicates that the regression model significantly predicts the dependent variable UR, and the predictors as a group have a meaningful impact on the outcome.^29^

**Table 6.** Analysis of Variance Test

Model	Sum of squares	df	Mean square	*F*	Significance
1					
Regression	75.492	3	25.164	59.394	0.000^a^
Residual	83.041	196	0.424		
Total	158.533	199			

^a^ Predictors: (Constant), LOR, LDL, and LT.

Dependent variable: UR.

LOR, lack of familiarity; LDL, lack of digital literacy; LT, lack of trust; UR, user resistance.

### Hypothesis test coefficients

The coefficients for the regression model examining the impact of LOR, LDL, and LT on UR were assessed (Table 7). The unstandardized coefficients revealed that LOR had a coefficient of 0.082 (standard error = 0.064), with a *t*-value of 1.283 and a *p* value of 0.201, indicating that LOR was not a statistically significant predictor of UR.

**Table 7.** Coefficients of Hypothesis Test

Model	Unstandardized coefficients		Standardized coefficients	*t*	Significance	95.0% confidence interval for *B*	
	*B*	Standard error	Beta			Lower bound	Upper bound
1							
(Constant)	0.525	0.275		1.906	0.058	−0.018	1.068
LOR	0.082	0.064	0.078	1.283	0.201	−0.044	0.207
LDL	0.350	0.056	0.359	6.261	0.000	0.240	0.461
LT	0.420	0.066	0.410	6.393	0.000	0.291	0.550

Dependent variable: UR.

LOR, lack of familiarity; LDL, lack of digital literacy; LT, lack of trust; UR, user resistance.

On the contrary, LDL had a significant positive effect on UR, with an unstandardized coefficient of 0.350 (standard error = 0.056), a *t*-value of 6.261, and a *p* value of <0.001, indicating strong statistical significance. Similarly, LT showed a significant positive relationship with UR, with an unstandardized coefficient of 0.420 (standard error = 0.066), a *t*-value of 6.393, and a *p* value of <0.001.

The 95% confidence intervals for the coefficients for LDL and LT were both above zero, further supporting the significance of these predictors. The confidence interval for LDL ranged from 0.240 to 0.461, and for LT, it ranged from 0.291 to 0.550.

The coefficients table provides detailed insight into the contribution of each independent variable—LOR, LDL, and LT—in predicting the dependent variable UR. The intercept (constant) is 0.525, which is the predicted value of UR when all predictors are zero; however, its *p* value is 0.058, indicating it is not statistically significant at the 0.05 level. Among the predictors, LDL and LT have significant positive effects on UR. The unstandardized coefficient for LDL is 0.350 (*p* < 0.001), meaning that for every one-unit increase in LDL, UR increases by 0.350 units, holding other variables constant. Similarly, LT has an unstandardized coefficient of 0.420 (*p* < 0.001), indicating a strong positive influence on UR. Both predictors have relatively high standardized beta coefficients (0.359 for LDL and 0.410 for LT), suggesting they are important predictors in the model. In contrast, LOR has a coefficient of 0.082, but it is not statistically significant (*p* = 0.201), suggesting that it does not have a meaningful impact on UR within this model. The 95% confidence intervals support these findings, as those for LDL and LT do not include zero, while the interval for LOR does (from −0.044 to 0.207), further indicating its nonsignificance.^29^

**FIG. 1.**
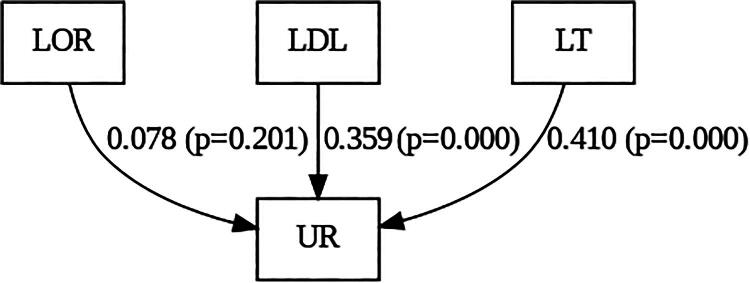
Path model showing user resistance factors. LOR, lack of familiarity; LDL, lack of digital literacy; LT, lack of trust; UR, user resistance.

A structural equation model was used to test the hypothesized relationships between LOR, LDL, LT, and UR to telemedicine. The path model, including standardized regression weights and significance levels, is shown in Figure 1).

The Kruskal–Wallis test shows that age influences resistance to telemedicine. The *p* value 0.008 is less than 0.05 (Table 8).^30^ So, the null hypothesis is rejected. This means there is a statistically significant difference in resistance to telemedicine (U1) across at least some age groups. However, the Kruskal–Wallis test does not tell us which groups are different. A post hoc pairwise comparisons are done to show which age groups differ.

**Table 8.** Kruskal–Wallis Test of Hypothesis

Null hypothesis	Test	Significant	Decision
The distribution of U1 is the same across categories of age.	Independent-samples Kruskal–Wallis test	0.008 (the significance level is 0.05)	Reject the null hypothesis

The post hoc pairwise comparison (Table 9) between 35–40 and 30–35 shows test statistics = 24.661, standard error = 8.738, standard test statistics = 2.822, significance = 0.005, adjusted significance = 0.048 (significant). People aged 35–40 show significantly higher resistance to telemedicine compared with those aged 30–35. The positive test statistic (24.661) indicates that 35- to 40-year-olds are more resistant than 30- to 35-year-olds. The post hoc pairwise comparison between 40 above and 30–35 shows test statistics = 30.656, standard error = 10.728, standard test statistics = 2.858, significance = 0.004, adjusted significance = 0.043 (significant). People aged 40 and above also show significantly more resistance to telemedicine than those aged 30–35. Again, the positive test statistic (30.656) means older individuals (40+) are more resistant than the 30–35 age group. All other comparisons (18–25 vs. 30–35, 25–30 vs. 30–35, etc.) have adjusted significance values well above 0.05, so they do not show statistically significant differences in resistance to adopting telemedicine (Table 9).^31^

**Table 9.** Post Hoc Pairwise Comparison Test

Sample 1 sample 2	Test statistics	Standard error	Standard test statistics	Significance	Adjusted significance
18–25 30–35	−38.032	15.600	−2.438	0.015	0.148
25–30 30–35	−27.801	11.538	−2.409	0.016	0.160
18–25 35–40	−13.370	15.291	−0.874	0.382	1.000
18–25 25–30	−10.231	17.047	−0.600	0.548	1.000
18–25 40 above	−7.376	16.510	−0.447	0.655	1.000
25–30 35–40	−3.140	11.117	−0.282	0.778	1.000
40 above 25–30	2.855	12.741	0.224	0.823	1.000
40 above 35–40	5.995	10.274	0.584	0.560	1.000
35–40 30–35	**24.661**	**8.738**	**2.822**	**0.005**	**0.048**
40 above 30–35	**30.656**	**10.728**	**2.858**	**0.004**	**0.043**

The bold lines show the age groups that significantly interact with the dependent variable UR and by this Hypothesis 4 has been supported.

### Hypothesis testing summary

The results of the hypothesis tests revealed varying degrees of significance in the predictors of UR. Hypothesis 1 proposed that an LOR would positively influence UR. However, this hypothesis was rejected as the relationship was not statistically significant (*p* = 0.201). This suggests that although users may be unfamiliar with telehealth platforms, LOR alone does not significantly deter adoption, particularly if users possess other skills, such as digital literacy and trust in the system.

In contrast, Hypothesis 2 posited that an LDL would positively influence UR, and this hypothesis was accepted. The relationship was found to be significant (*p* < 0.001), indicating that users who perceive themselves as digitally unskilled are more likely to resist adopting telehealth. Digital literacy thus plays a crucial role in facilitating effective engagement with telehealth technologies.

Similarly, Hypothesis 3 suggested that an LT would positively influence UR, which was also accepted. The results showed that trust concerns, particularly regarding data privacy, diagnosis accuracy, and the security of telehealth platforms, were significant barriers to adoption (*p* < 0.001). Users need strong assurances regarding the handling of their data and the reliability of the technology to overcome these concerns.

Hypothesis 4 proposed that women’s resistance to telemedicine would significantly differ across age groups. Age plays a meaningful role in influencing resistance to telemedicine among women, with mid-to-older age groups exhibiting higher levels of resistance compared with younger adults.

## Discussion

The purpose of this study was to assess the psychological factors influencing UR to adopting telemedicine technology by testing three key predictors: LOR, LDL, and LT. Based on the findings from the regression analysis, several key insights emerged that shed light on the dynamics of resistance toward telemedicine systems.

The overall regression model was statistically significant (*F* = 59.394, *p* < 0.001), explaining 47.6% of the variance in women’s resistance to adopt telemedicine technologies. This suggests that psychological barriers play a substantial role in shaping women’s attitudes toward adopting telehealth platforms.

LT and LDL emerged as significant predictors of UR to telehealth. Among them, LT had the strongest standardized beta coefficient (β = 0.410), closely followed by LDL (β = 0.359), indicating that women are most concerned with the security, privacy, and reliability of telehealth services. These results are consistent with existing literature that emphasizes the critical role of trust in technology adoption—particularly in health care, where the perception of risk, such as data security and privacy concerns, can strongly influence behavior. Studies found that trust in the health care provider, platform, and technology infrastructure must be established before patients feel comfortable using telemedicine as a substitute for face-to-face care.^32^ Similarly, LDL—defined as the limited ability to navigate, understand, and interact with digital tools—has been found to strongly deter users from engaging with telehealth platforms.^7^

LDL was also a significant determinant, reinforcing the notion that many users feel unprepared or lack the confidence to engage with telehealth systems. The need for digital training, simplified interfaces, and user-friendly platforms is crucial,^13^ especially for older adults or those with limited exposure to digital technologies. Without digital literacy, women may struggle to navigate telehealth platforms, making them more likely to resist adoption.

Interestingly, LOR did not significantly predict women resistance to telehealth in this model (*p* = 0.201). While initial assumptions suggested that unfamiliarity could lead to discomfort or avoidance, the data indicate that mere unfamiliarity may not be enough to deter adoption unless it is accompanied by low digital skills or concerns about trust. This could suggest that women might be unfamiliar with telehealth but still open to learning—if the platforms are perceived as secure and the interfaces are user-friendly. Studied found that perceived familiarity was a critical adoption factor where users unfamiliar with mHealth applications reported significantly lower intention to use them. This LOR includes limited knowledge of how to access telehealth services, what services are available, or how to evaluate their credibility.^33^

Furthermore, the reliability of the scales used was strong across all constructs, with Cronbach’s α values exceeding 0.70, indicating that the questionnaire items consistently measured their intended psychological factors.

This study found that resistance to telemedicine was significantly higher among women aged 35–40 and 40 and above, compared with younger age groups. This finding is aligned with previous research indicating that older women often exhibit greater hesitancy toward digital health technologies due to lower digital confidence, lack of exposure, and concerns about data privacy.^8^

These findings have important implications for telehealth providers, developers, and policymakers. Efforts to promote telehealth adoption should prioritize building trust through transparent communication about data security, privacy, and system reliability while also investing in digital literacy programs that empower users to interact confidently with telehealth technology.

## Conclusion

This study highlights the psychological barriers contributing to UR in adopting telemedicine technologies, specifically focusing on LOR, LDL, and LT. Among these, LDL and LT were the most significant predictors of resistance, accounting for nearly half of the variance in user attitudes toward telehealth adoption. While LOR was hypothesized to influence resistance, it did not show statistical significance, suggesting that users may be unfamiliar but not necessarily resistant—unless they also lack digital skills or harbor distrust. These insights underscore the critical need for targeted interventions to enhance trust and improve digital competencies among telehealth users. By addressing these psychological barriers along with older women’s age groups, stakeholders can facilitate smoother transitions to telehealth platforms, ultimately leading to broader adoption and more inclusive health care access in the digital age.

### Implications

The results have practical implications for telehealth providers, tech developers, and policymakers. To increase telehealth adoption, strategies should prioritize:

- Building user trust through transparent privacy policies and clear communication of data protection practices to alleviate concerns about the security and reliability of telehealth platforms.
- Enhancing digital literacy through training sessions, simplified user interfaces, and support services tailored to different user groups (especially older adults or low-education users). This would equip users with the confidence and skills needed to engage with telehealth technology.
- Recognizing that initial unfamiliarity with telehealth may not be a significant barrier by itself. However, addressing underlying concerns related to trust and usability is essential for fostering adoption.

### Limitations of the study

The study concentrated solely on psychological factors (LOR, LDL, LT) and did not account for other important variables such as technological infrastructure, institutional readiness, or cultural influences. All the respondents were women from different age groups. Sample size can be further expanded, including men or other, to identify the impacting factors of resistance to adopt telemedicine technologies.

### Further research areas

Building upon the findings and limitations of this study, future research could explore the following areas:

- **Longitudinal studies:** Tracking how user attitudes and resistance to telehealth change over time, especially after interventions aimed at building trust or improving digital skills.
- **Broader demographic and regional representation:** Expanding the study to include participants from diverse age groups, educational backgrounds, and both urban and rural areas across the country for a more comprehensive analysis.
- **Inclusion of technological and institutional factors:** Investigating the role of technological barriers (e.g., internet access, device availability) and institutional support (e.g., policy implementation, health care staff training) in influencing telehealth adoption.
- **Experimental interventions:** Designing and testing targeted interventions (e.g., digital literacy programs, trust-building campaigns) and measuring their impact on reducing resistance.
- **Health care provider perspectives:** Exploring how the attitudes and digital readiness of health care professionals influence the promotion and success of telehealth initiatives.

By considering these additional areas, future research can provide a more holistic understanding of the factors influencing telehealth adoption and help to create more effective strategies for overcoming barriers.

## Abbreviations Used

LDL: Lack of digital literacy
LOR: Lack of familiarity
LT: Lack of trust
UR: User resistance
